# Synthesis and characterization of novel acrylamide derivatives and their use as corrosion inhibitors for carbon steel in hydrochloric acid solution

**DOI:** 10.1038/s41598-023-30574-3

**Published:** 2023-03-02

**Authors:** A. S. Fouda, E. M. Khalil, G. A. EL-Mahdy, M. M. Shaban, A. S. Mohammed, N. A. Abdelsatar

**Affiliations:** 1 Deparment of Chemistry, Faculty of Science, Mansoura University, Mansoura, 35516 Egypt; 2 Department of Chemistry, Faculty of Science, Helwan University, Cairo, Egypt; 3 Applied Surfactant Lab, Egyptian Petroleum Research Institute, Nasr City, Cairo Egypt; 4 Refining and Processing Deputy, Egyptian General Petroleum Corporation, Cairo, Egypt; 5 Department of Chemistry, Faculty of Science, Ain Shams University, Cairo, Egypt

**Keywords:** Corrosion, Materials science

## Abstract

Two new acrylamide derivatives were prepared namely: “N-(bis(2-hydroxyethyl) carbamothioyl) acrylamide (BHCA) and N-((2-hydroxyethyl) carbamothioyl) acrylamide( HCA) and their chemical structures were analyzed and confirmed using IR and 1H NMR”. These chemicals were investigated as corrosion inhibitors for carbon steel (CS) in 1 M HCl medium using chemical method (mass  loss, ML), and electrochemical techniques including potentiodynamic polarization (PDP), and electrochemical impedance spectroscopy (EIS). The results showed that the acrylamide derivatives work well as corrosion inhibitors, with inhibition efficacy (%IE) reaching 94.91–95.28% at 60 ppm for BHCA and HCA, respectively. Their inhibition depends mainly on their concentration and temperature of the solution. According to the PDP files, these derivatives function as mixed-type inhibitors that physically adsorb on the CS surface in accordance with the Langmuir adsorption isotherm, creating a thin coating that shields the CS surface from corrosive fluids. The charge transfer resistance (R_ct_) increased and the double layer capacitance (C_dl_) decreased as a result of the adsorption of the used derivatives. Calculated and described were the thermodynamic parameters for activation and adsorption. Quantum chemistry computations and Monte Carlo simulations were examined and discussed for these derivatives under investigation. Surface analysis was checked using atomic force microscope (AFM). Validity of the obtained data was demonstrated by the confirmation of these several independent procedures.

## Introduction

Corrosion is the degradation of a material caused by its interaction with other materials and/or the environment^1^. CS plays an important role in many industries; particularly the oil and gas industry^2–5^. It is the most widely used engineering material, accounting for approximately 85% of annual steel production worldwide. CS corrosion is one of the most serious issues that many industries face, particularly in the oil and gas industry, because it not only affects production stability but also negatively impacts project economics. The importance of the study of CS corrosion in acidic media especially hydrochloric acid comes out because of the spreading of the industrial applications of acid solutions. Corrosion problems in refining industries are attributed to the acidic additives which the equipment exposed to surface^6^ . Also, acid pickling, acid cleaning, acid descaling and oil recovery are considered to be other applications for acid in many industries^7^. Steel exposure to corrosive environments leads to different kinds of corrosion mechanisms; so, the application of corrosion inhibitors to resist metal degradation will be mandatory^8^. The corrosion of metallic items in an acidic solution actually results in significant expense^9^. The most commonly used corrosion inhibitors are organic derivatives with heterocyclic atoms such as O, N, and/or S atoms in their structure^10^. Abu-Rayyan et al.^11^ synthesized some acrylamide derivatives namely: “2-cyano-N-(4-hydroxyphenyl)-3-(4-methoxyphenyl) acrylamide (ACR-2) and 2-cyano-N-(4-hydroxyphenyl)-3-phenylacrylamide (ACR-3)” and used them as corrosion inhibitors for Cu in 1 M HNO_3_ and obtained maximum efficiencies of 84.5% and 86.1%, respectively at 20 × 10^−5^ M. Novel acrylamide ionic liquids as anti-corrosion for X-65 steel dissolution in acid medium was reported by El-Tamany et al.^12^. Zaki et al. utilized methyl acrylate derivatives: named 2-Methacryloyloxyethyloctadecyldimethyl ammonium bromide (MEODAB), poly (2-Methacryloyloxyethyl octadecyldimethyl ammonium bromide) (PMEODAB) as corrosion inhibitors for X-65 type CS in 1 M HCl^13^. Amides and derivatives like urea, thiourea or thioacetamide display satisfactory performances as inhibitors for mild steel in acid solutions^14,15^. Because the prepared acrylamide compound contains O, N, and/or S atoms, which allow adsorption on the metal surface^16–19^, the overlapping of P orbitals of hetero atoms with empty d orbitals of metal increases the possibility of using the prepared acrylamide compound as a corrosion inhibitors^20–25^. Some acrylamide derivatives were utilized as corrosion inhibitors for metals in altered acid solutions with their percentage inhibition (%IE) were predicted in Table 1.

**Table 1:** List of acrylamide derivatives used for the corrosion inhibition of metals in altered acid medium.

Compound	Sample	Medium	%IE	Ref.
a) poly-2-acrylamido-2-methylpropane sulfonic acid triethanolamine derivative (P1) b) poly-2-acrylamido-2-methylpropane sulfonic acid triethylamine derivative (P2) c) poly-2-acrylamido-2-methylpropane sulfonic acid trimethylamine derivative (P3)	X65 Steel	1 M HCl	91.4%, 83.7% and 80.0%, at 250 ppm, respectively	^29^
a)N,N-bis(2-hydroxyethyl) acrylamide (DEA) b)N-(2-hydroxyethyl) acrylamide (MEA)	Carbon steel	1 M HCl	94.1% and 93.6%, at 60 ppm respectively	^30^
poly(2-acrylamido-2-methyl-1-propane-sulfonic acid-co–N-isopropyl acrylamide) hydrogels	Steel	1 M HCl	94% at 10^−3^ M	^31^
PAMPS-Na-co-St/magnetite composite	Steel	1 M HCl	99% at 250 ppm	^32^
a) Pectin-g-polyacrylamide (denoted as Pec-g-PAAm) b) pectin-g-polyacrylic acid (denoted as Pec-g-PAA)	Mild Steel	3.5% NaCl	85.53% at 800 ppm 75.48% at300 ppm, respectively	^33^
a)N-(bis(2-hydroxyethyl) carbamothioyl) acrylamide (BHCA) b)N-((2-hydroxyethyl) carbamothioyl) acrylamide (HCA)	API 5 l X52 Carbon Steel	1 M HCl	95.3%, 94.9% at 60 ppm, respectively	Our result

Two different electrochemical techniques [potentiodynamic polarization (PDP) and electrochemical impedance spectroscopy (EIS)] were used to investigate the efficacy of the prepared derivatives at 298 K to resist the corrosion of CS in 1 M HCl solution, while the mass loss method was used at different temperatures ranging from 298 to 318 K, in addition to the use of quantum chemical calculations and Monte Carlo simulation. Also, the surface analysis was performed using atomic force microscope (AFM). In this study, Acryloyl chloride base was reacted with ammonium thiocyanate then the product will react in equal amount with ethanolamine to produce the studied organic corrosion inhibitors named: N-((2-hydroxyethyl) carbamothioyl) acrylamide (HCA) and N-(bis (2-hydroxyethyl) carbamothioyl) acrylamide (BHCA) which were examined and proved through the usage of both IR, 1HNMR techniques, and because the produced acrylamide compounds contain O, N, and/or S atoms, which enable adsorption on the metal surface^26^, there is a greater chance that they will be utilized as corrosion inhibitors when the p orbitals of hetero atoms and vacant d orbitals of metal overlap^27^. The chemicals' contact with the corroded metal surface can also result in the formation of a thin protective layer on the metal surface^28^.

The goal of this study is to determine the effectiveness of two synthetic compounds, N-((2-hydroxyethyl) carbamothioyl) acrylamide (HCA) and N-(bis (2-hydroxyethyl) carbamothioyl) acrylamide (BHCA), against the dissolution of CS in an acidic medium (1 M hydrochloric acid solution, using chemical and electrochemical methods as well as characterization techniques from Monte Carlo simulations, density functional theory (DFT) and AFM, to discover the adsorption type and corrosion mechanism on the CS surface.

## Materials and methods

### Materials

**Figure 1:**
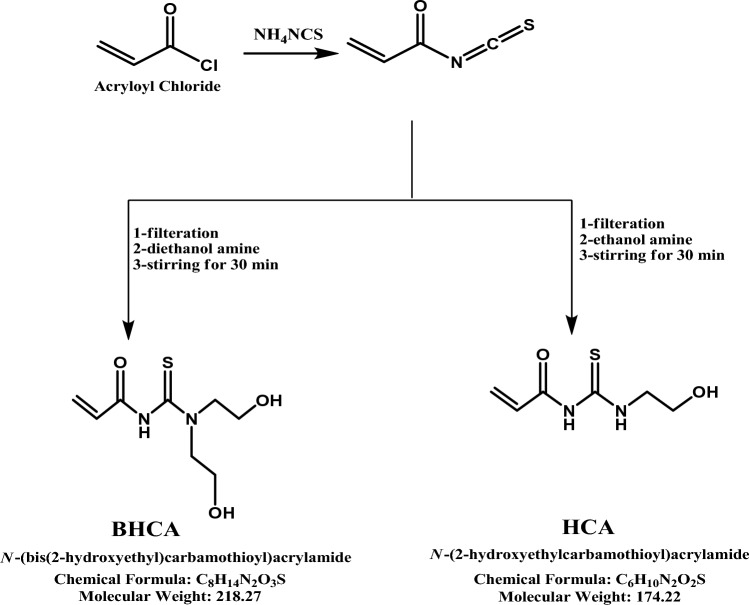
Chemical structure of the synthesized HCA and BHCA derivatives.

1. *Carbon steel API 5L X52* with its composition was reported as: 0.16% Carbon, 0.45% Silicon, 1.65% Manganese, 0.02% Phosphorus, 0.01% Sulphur, 0.07% Vanadium, 0.05% Niobium, 0.04% Titanium and Fe as balance.
2. *Chemical product used* Acryloyl chloride, Ethanolamine, Diethanolamine all BDH grade and all Purchased from Al-Gomhoria Company, Egypt. The synthesized acrylamide derivatives (HCA and BHCA) with their molecular formulas and molecular weights are shown in Fig. 1
3. *Hydrochloric Acid* was purchased from Al-Gomhoria Company, Egypt. In every experiment, the corrosive medium was a solution of 1 M HCl. A 37% HCl solution of Analar grade was diluted to create the corrosive solutions.

## Synthesis of acrylamide derivatives: (N-((2-hydroxyethyl) carbamothioyl) acrylamide (HCA) and N-(bis (2-hydroxyethyl) Carbamothioyl) acrylamide (BHCA)^34^

The reaction of “Acryloyl chloride (0.9 g, 100 mmol) with ammonium thiocyanate (0.76 g, 100 mmol)in dry acetone with stirring for 10 min, furthermore, the ammonium chloride was filtered off and the reaction mixture was stirred with different amines namely Diethanolamine and ethanolamine (100 m moles), respectively for 30 min, then acetone was evaporated and wash the product with petroleum ether to remove the unreacted derivatives the remaining oil is the product (HCA) and (BHCA), respectively”, The formed precipitate was filtered off, dried.

### Derivative HCA

Light brown liquid, 89% yield, “IR (KBr, ν /cm^−1^): broadband located at 3281.8 cm^−1^ (νOH), 3281.8, 3120.5 cm^−1^ (νNH); 1690.2 cm^−1^ (νC = O). 1H-NMR (300 MHz, DMSO-d6): δ 2.2 (t, 2H, H next to OH), 2.5 (t, 2H, H next to N), 3.5 (dd, 1H, CH(b)), 4.2 (q, 1H, CH(a)), 6.2 (dd, 1H, CH(c)), 7.8 (s, 1H, NH, D2O exchangeable), 8.0 (s, 1H, NH next to CO, D2O exchangeable), 8.1 (s, 1H, 1OH, D2O exchangeable). The chemical structure of the prepared compound was confirmed using elemental analysis and the result is shown in Table 2”.

**Table 2:** Elemental analysis of the synthesized HCA and BHCA.

Comp.,	M.Wt g/mol	Mol. Formula	C%		H%		N%		O%		S%	
			calculated	found	calculated	found	calculated	found	calculated	found	calculated	found
HCA	174.22	C_6_H_10_N_2_O_2_S	41.36	41.20	5.79	5.82	16.08	15.98	18.37	18.33	18.40	18.13
BHCA	218.27	C_8_H_14_N_2_O_3_S	44.02	44.10	6.46	6.44	16.08	16.10	21.99	21.87	14.69	14.60

### Derivative BHCA

**Figure 2:**
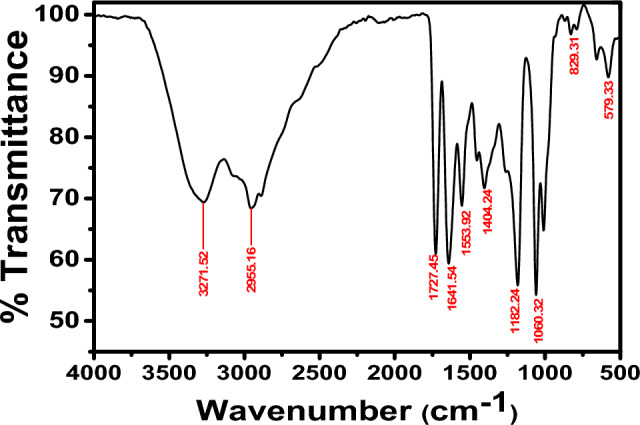
IR (KBr) for the two derivatives.

Dark brown liquid, 82% yield, “IR (KBr, ν /cm^−1^): as shown in Fig. 2 broadband located at 3336.9 cm^−1^ (νOH), 3336.9 cm^−1^ (νNH); 1704.6 cm^−1^ (νC = O). 1H-NMR (300 MHz, DMSO-d6): δ 2.0 (s, 1H, NH, D2O exchangeable), 2.4 (t, 2H, H next to OH), 2.5 (dd, 1H, CH(b)), 2.9 (t, 2H, H next to N), 3.1 (q, 1H, CH(a)), 3.6 (dd, 1H, CH(c)), 6.2 (s, 2H, 2OH, D2O exchangeable)., The chemical structure of the prepared compound was confirmed using elemental analysis and the result is shown in Table 2”.

### Methods

#### Mass loss (ML) tests

ML tests are the most accurate and precise method for determining metal corrosion rate because the experiment is simple to replicate and, even with long exposure times, the results are consistent, “the relatively simple procedure reduces the propensity to introduce systematic errors^35^. On seven CS samples, ML tests were taken. The seven identical specimens, each measuring 2.0 × 2.0 × 0.2 cm, were abraded to different degrees of sandpaper, degreased with acetone, and stored in a desiccator. At 298 K, the seven specimens were immersed in 100 mL hydrochloric acid (1 M) without and with varied concentrations of the investigated inhibitors (10, 20, 30, 40, 50, and 60 ppm by weight). After varying immersion time intervals of 30, 60, 90, 120, 150, and 180 min”, the specimens were rinsed, good drying, and weighed properly.

The following Eq. (1) was used to calculate the efficacy of inhibition (percent IE) of examined chemicals for metal corrosion in 1 M HCl^36^:

1 $$\% \;IE = 100 \times \theta$$

The degree of surface coverage (${\uptheta })$ is computed using the equation below (2)^36^.

2 $$\theta = \left( {1 - \frac{\Delta W}{{\Delta W^\circ }}} \right)$$

where ΔW and ΔW° are MLs per unit area (mg cm^−2^) in the presence and the absence of the tested inhibitors, respectively^37^.

#### Electrochemical tests

Three different electrochemical techniques were applied to study the corrosion characteristics of “API 5L X52 CS in 100 ml of 1 M HCl in the presence and the absence of different concentrations of the investigated HCA and BHCA derivatives ranged from 10 to 60 ppm, all of these approaches were carried out at 298 K in a typical glass cell with three electrode systems: a CS specimen with a surface area of 1 cm^2^, a saturated calomel electrode (SCE) as a reference electrode, and a counter or auxiliary electrode made of platinum. The working electrode is prepared by welding the CS specimen with a copper rod from one side and perfectly isolated into a glass rod so that one face of CS was left to be exposed to the test solution^38^. The temperature of the cell was organized by a water bath. Before starting the experiments, the CS specimen is mechanically abraded with different degrees of sandpaper and degreased using acetone then rinsed with bi-distilled water and finally dried between filter paper. The working electrode was immersed in the tested corrosive solution until the open circuit potential was reached in min approximately. Volta lab 40 (Tacussel-radiometer PGZ301) was used for all electrochemical measurements and computations, which were controlled using the Tacussel corrosion analysis software model (Volta master4)^39^. Data fitting, graph sketching, and plotting are all possible with Echem software 6.03”.

For the measurement of polarization value, experiments were carried out at 1 mVs^−1^and within the range of ± 250 mV. The density of the corrosion current is achieved by extrapolation of anodic and cathodic Tafel lines to a point which provides log i_corr_ and the equivalent corrosion potential (E_corr_) for compound free solution and for each concentration of the prepared HCA and BHCA derivatives. (%IE) and (θ) were measured according the following Eq. (3)^40,41^:

3 $$\% \;IE = 100 \times \theta = [1 - \, \left( {i_{corr} / \, i^{o}_{corr} } \right] \times 100$$

where i_corr_ and i^o^_corr_ are the intensity of corrosion current in the presence and the absence of the tested derivatives, respectively.

EIS measurements at open circuit potential and in a frequency range of 100 kHz to 0.5 Hz were used to characterize the kinetics of electrochemical processes and the capacitive behavior of investigated derivatives on CS. As demonstrated in Eq. (4)^42^, charge transfer resistance derived from EIS data was utilized to compute percent IE” and (θ).

4 $$\% \;IE = 100 \times \theta = \left[ {1 - \left( {R^{o}_{ct} /R_{ct} } \right)} \right] \, \times 100$$

where R^o^_ct_ and R_ct_ are the resistance of the charge transfer in the absence and presence of the tested derivatives, respectively.

### Surface analysis

The AFM analysis was bused to observe the surface of CS immersed for 24 h in 1 M HCl solution in the presence of inhibitors using a picoSPM2100 AFM device operating in contact mode in air.

### Theoretical studies

#### Quantum chemical calculations

The PM3 semi-empirical method was used to optimize the molecular structures of the prepared HCA and BHCA organic inhibitors. Spartan 10 V1.10 was used to perform all of the quantum's chemical calculations.

#### Monte Carlo simulations

Because it includes certain crucial factors such as total energy, adsorption energy and stiff adsorption energy, Monte Carlo simulations are thought to be one of the most widely used theoretical tools to understand the interaction between metal and inhibitor^43–45^. In the current study, the lowest energy for the tested system was determined using a Monte Carlo simulation calculation. “The results and descriptors obtained through Monte Carlo simulations, including the total adsorption, adsorption energy, firm adsorption, and deformation energies that represent the most stable low energy configuration for the adsorption of the investigated HCA and BHCA inhibitors on Fe (110) surface”.

## Results and discussion

### ML tests

**Figure 3:**
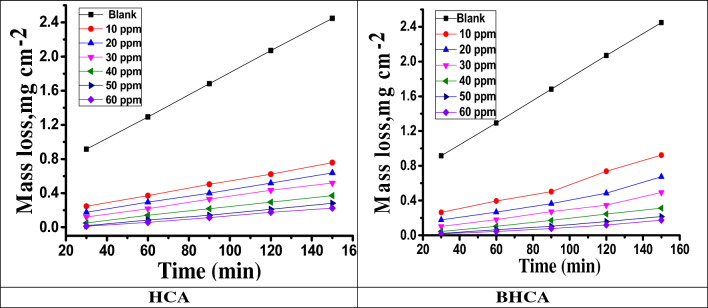
ML-time curves of CS with and without various concentrations of HCA and BHCA in 1 M HCl.

Because of its ease of use and excellent dependability, it is a particularly valuable tool for evaluating and monitoring corrosion rate and inhibition efficacy^46^. Table 3 illustrates the inhibitors' inhibition efficiency and surface coverage (θ) for CS corrosion after 120 min in 1 M HCl at 298 K. It was observed that the corrosion of CS is inhibited because of using the studied HCA and BHCA inhibitors and it was noticed also that the inhibition efficiencies increase by increasing the inhibitors concentration because of increasing of the coverage area of CS surface by the inhibitors. The ML—time curves for CS dissolution in 1 M HCl in the presence and absence of different concentrations of the tested inhibitors HCA and BHCA are also shown in Fig. 3.The inhibition of the corrosion reaction rate is due to adsorption of the HCA and BHCA inhibitor molecules forming a protective layer on the metal surface, which suppress the interaction between acidic medium and metal surface^47^. As shown in Table 3 the maximum %IE obtained in the presence of BHCA inhibitor is 91.7% at 60 ppm and 90.9% at 60 ppm for HCA. As the concentration increases, the area of the metal surface covered by the inhibitor molecule also increases, leading to an increase in the %IE and decrease ML. Because of the continuous accumulation of inhibitor molecules on the metal surface, which increases the thickness and density of the adsorbed protective layer and thus increases the %IE^48^, it appears that increasing the inhibitor concentration and the immersion time of CS specimens in HCl solution reduces corrosion rates. The order of the prepared inhibitors based on %IE is: BHCA > HCA.

**Table 3:** illustrates the inhibitors' inhibition efficiency for CS corrosion after 120 min in 1 M HCl at 298 K.

Compound	Concentration, ppm	%IE
HCA	10	70.2
	20	79.3
	30	83.6
	40	87.0
	50	90.1
	60	90.9
BHCA	10	76.4
	20	82.5
	30	85.3
	40	88.4
	50	90.6
	60	91.7

### Adsorption isotherm

The quantitative expression of the adsorption processes for the prepared HCA and BHCA inhibitors on the metal surface, the inhibition pathway can be determined via the isotherm of^49^. “It is also possible to see the inhibitor and the metal interaction via isotherm diagrams. The adsorption of inhibitors at the metal–solution interface is represented as a substitution adsorption process between the inhibitor molecules Inh_(sol)_ and the water molecules on metal surface (H_2_O_ads_)”:

5 $${\text{Inh}}_{{({\text{sol}})}} + {\text{nH}}_{{2}} {\text{O}}_{{{\text{ads}}}} \to {\text{Inh}}._{{({\text{ads}})}} + {\text{H}}_{{2}} {\text{O}}_{{({\text{sol}})}}$$

**Figure 4:**
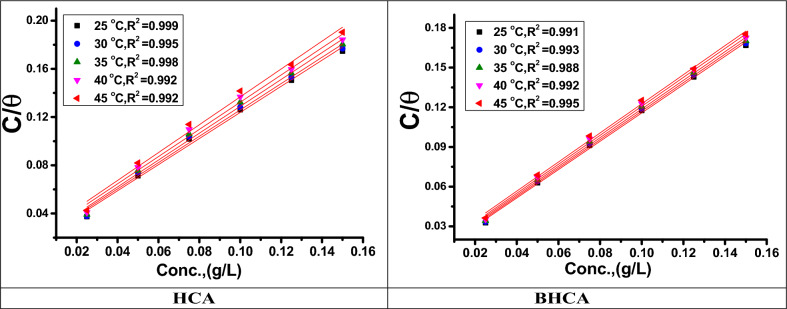
“Langmuir isotherm plots as C/θ vs. C, of HCA & BHCA for dissolution of CS in 1 M HCl at various temperatures”.

“Where Inh_(sol)_ and Inh_(ads)_ are the dissolved inhibitor molecules in the corrosive solution and the adsorbed inhibitor molecules onto the metal surface, respectively. While H_2_O_(ads)_ is the adsorbed molecules of water on the metal surface and n is the ratio which reflects the number of water molecules replaced by each inhibitor molecule. To determine the most suitable diagrams several adsorption isotherms (Langmuir, Temkin, Freundlich) were applied for the description of adsorption of the prepared HCA and BHCA inhibitors on CS surface, but the best fit was Langmuir isotherm as shown in Fig. 4. (The value of the correlation coefficient approached the right one) which can express as shown in Eq. (6)^50^:

6 $$\frac{C}{\theta } = \frac{1}{Kads} + {\text{C}}$$

where C is the inhibitor molar concentration, θ is the coverage surface degree ($\theta = \frac{{\%{\text{IE}}}}{100}$)^51^ and K_ads_ is the adsorption equilibrium constant. Straight lines obtained by plotting $\frac{{\text{C}}}{{\uptheta }}$ versus C (Fig. 3) gives intercept with a value equal to 1/K_ads_ . In Langmuir model, the relationship between K_ads_ and ΔG^o^_ads_ can be calculated as expressed in Eq. (7)^52^:

7 $$55.5\;{\text{K}}_{{{\text{ads}}}} = {\text{exp}}\left[ { - \Delta {\text{G}}^{{\text{o}}}_{{{\text{ads}}}} /{\text{RT}}} \right]$$

where 55.5 is the molar concentration of water in the solution M^−1^. For the manufactured HCA and BHCA, the values of ΔG^o^_ads_ were determined to be (21.7–18.7) and (22.9–18.8) kJ mol^−1^, respectively, as shown in Table 4. In general, ΔG^o^_ads_ values around − 40 kJ mol^−1^ or above imply chemisorption, while values of − 20 kJ mol^−1^ or below indicate physisorption interaction between the inhibitor molecule and the metal surface^53,54^. Negative values of ΔG^o^_ads_ show the spontaneity of the adsorption process and the durability of the adsorbed layer of the produced derivatives on CS surface, whereas negative values of ΔH^o^_ads_ indicate the presence of chemical and physical adsorption^55^. Chemisorption enthalpy levels can be as high as about100 kJ mol^−1^, while physisorption enthalpy levels, which occur from electrostatic interactions between charged molecules and charged metals, can be as high as 41.9 kJ mol^−1^. Absolute enthalpy values of physisorption-inducing molecules are low. When the investigated chemicals are present, the ΔS^o^_ads_ values are large and negative, which denotes an increase in the level of ordering on the CS surface^56^.

**Table 4:** Adsorption parameters of HCA and BHCA on CS substrate in 1 M HCl.

Inh	Temp., K	Adsorption parameter				
		R^2^	K_ads_ M^−1^	−∆G^°^_ads_ kJ mol^−1^	−∆H^°^_ads_ kJ mol^−1^	−∆S^°^_ads_ J mol^−1^ K^−1^
HCA	298	0.999	66	21.7	27.6	66.2
	303	0.995	61	21.0		65.5
	313	0.998	57	19.7		64.3
	323	0.992	49	19.5		62.3
	333	0.992	46	18.7		60.5
BHCA	298	0.991	115	22.9	24.5	69.5
	303	0.993	108	22.1		68.1
	313	0.988	100	21.2		65.2
	323	0.992	91	20.3		64.8
	333	0.995	75	18.8		62.1

### Effect of temperature

In the range of temperatures 298 to 318 K, the effect of temperature on the rate of corrosion on CS was investigated. It was discovered that as the temperature rises, the corrosion rate increases and the inhibition efficacy decreases, indicating that the adsorption behavior is physisorption^56^.

### Kinetic parameters for corrosion process

**Figure 5:**
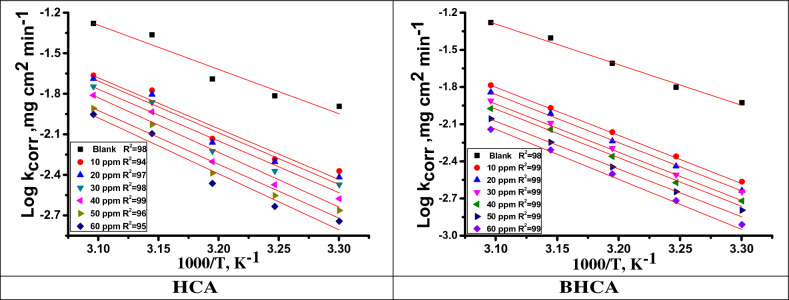
Arrhenius plots for dissolution of CS in 1 M HCl without and with of altered concentrations of HCA &BHCA.

The effect of temperature on the corrosion rate of CS in 1 M HCl solution with various concentrations of the studied HCA and BHCA inhibitors was studied using mass loss tests over a temperature range of 298 to 318 K. The data revealed that the rate of corrosion increases as the temperature rises^56^. Plotting log k_corr_ (corrosion rate) versus 1/T (absolute temperature, K) for API 5LX52 CS in HCl yielded straight lines as shown in Fig. 5 for HCA and BHCA. Using the Arrhenius Eq. (8), the value of the Arrhenius activation energy (E^*^_a_) can be computed (Fig. 4).

8 $$k_{corr} = \, A \, exp \, \left( {-E^{*}_{a} / \, RT} \right)$$

**Figure 6:**
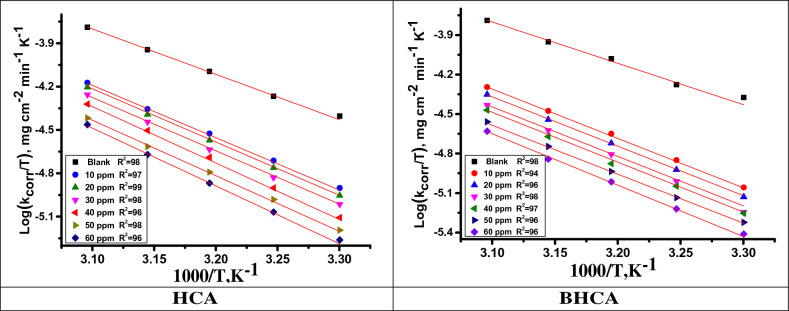
Corrosion of CS in 1 M HCl in the absence and presence of altered concentrations of HCA &BHCA plotted as (log k_corr_/T) vs. 1000/T.

The absolute temperature is T, the Arrhenius constant is A, the rate of corrosion is k_corr_, and the universal gas constant is R. Equation (9) can be used to calculate the activation (∆S^*^) entropy and (∆H^*^) enthalpy:

9 $$k_{corr} = \, \left( {RT/Nh} \right)exp \, \left( {\Delta S^{*} /R} \right)exp\left( { - \Delta H^{*} / \, RT} \right)$$

where h represents Planck's constant, N represents Avogadro's number, ∆S^*^ represents activation entropy, and ∆H^*^ represents activation enthalpy. When plotting log (k_corr_ /T) vs 1000/ T, straight lines with a slope equal to (∆H^*^/2.303R) and intercept equal to [log (R/Nh + ∆S^*^ / 2.303R)] are produced, as illustrated in Fig. 6 for HCA and BHCA, respectively. The rise in E^*^_a_ in the presence of prepared HCA and BHCA is due to physisorption between the inhibitor molecules and the CS surface, as demonstrated in Table 5. Higher E^*^_a_ values result in a lower rate of corrosion. This is because a thin coating forms on the CS surface, which acts as an energy barrier to CS corrosion. The rate-determining step, activated complex, has a negative entropy value, indicating that it is an association phase rather than a dissociation step^57^.

**Table 5:** Activation parameters for dissolution of CS in 1 M HCl in the absence and with altered amounts of HCA &BHCA.

Inhibitor	Conc., ppm	Kinetic Activation parameters		
		E_a_* kJ mol^−1^	−ΔH* kJ mol^−1^	−ΔS* J mol^−1^ K^−1^
Blank	0.0	62.6	60.0	85.8
HCA	10	71.9	69.7	82.8
	20	73.5	70.8	63.1
	30	74.3	71.2	61.9
	40	74.9	72.3	61.4
	50	75.5	73.4	59.6
	60	77.4	74.7	59.2
BHAC	10	73.5	70.9	80.9
	20	74.4	71.8	58.8
	30	75.3	72.7	58.4
	40	76.2	73.6	56.8
	50	77.5	74.9	55.8
	60	79.1	76.5	55.9

### Electrochemical measurements

#### PDP tests

**Figure 7:**
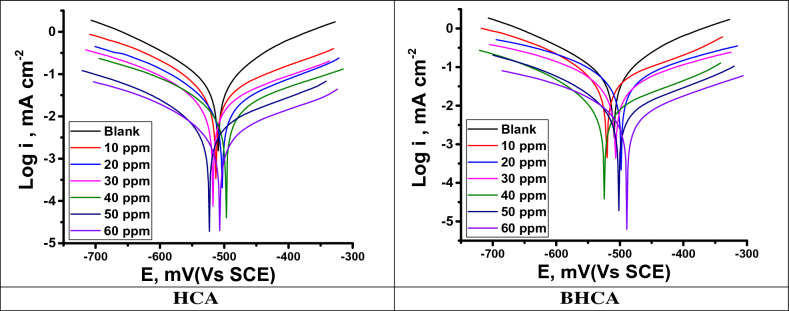
PDP bends for the dissolution of CS in 1 M HCl with and without altered doses of HCA &BHCA at 298 K.

As illustrated in Fig. 7 the anodic and cathodic interactions are tightly restricted based on the kinetics of the cathodic and anodic reactions in the presence and absence of the changed concentrations of HCA and BHCA derivatives. Additionally, the parallel Tafel lines after the addition of the inhibitors and the blanks demonstrate that the inclusion of the inhibitors did not alter the reaction's mechanism^58^. Table 6 displays the parameter values of the corrosion potential (E_corr_), anodic and cathodic slopes (ß_a_, ß_c_), and corrosion current (i_corr_). It can be seen that the addition of various concentrations of BHCA and HCA causes a significant decrease in the values of i_corr_, and that this increase causes a decrease in the current density to the bare minimum^59^. Both derivatives BHCA and HCA can provide a highly effective inhibitory performance for CS in hydrochloric acid due to the adsorption of a layer of the inhibitor on the metal's surface, which supports their ability to increase the values of the inhibition coefficient. Additionally, a slight variation in E_corr_ values supports the mixed nature of these compounds^60^. According to this study’s findings, the inhibitors under examination are mixed type inhibitors with a maximum displacement in E_corr_ of 47 mV^61^. The order of the prepared inhibitors based on IE% is BHCA > HCA.

**Table 6:** PDP data of CS in 1 M HCl with and without altered doses of HCA and BHCA at 298 K.

Inhibitor	Conc., ppm	−E_corr_ mV (vs SCE)	i_corr_ μA cm^−2^	*β*_a_ mVdec^−1^	*− β*_c_ mVdec^−1^	*R*_*p*_ (Ω cm^2^)	Θ	% IE
Blank	–	509.2	638.1	107.2	149.5	42.48	–	–
HCA	10	512.9	349.2	128.9	191.4	80.54	0.453	45.3
	20	503.20	270.4	110.9	167.3	104.76	0.576	57.6
	30	517.6	213.5	87.8	145.6	125.97	0.665	66.5
	40	496.5	118.2	100.4	158.7	251.96	0.815	81.5
	50	523.1	69.1	134.3	175.2	455.61	0.892	89.2
	60	506.8	32.5	142.4	185.6	706.27	0.949	94.9
BHCA	10	500.6	325.8	114.3	177.8	87.91	0.489	48.9
	20	529.4	246.8	130.2	161.7	108.54	0.613	61.3
	30	512.5	187.1	89.5	163.3	178.26	0.707	70.7
	40	551.7	114.3	145.7	187.2	261.82	0.821	82.1
	50	520.3	54.7	137.9	111.7	483.37	0.914	91.4
	60	504.1	30.1	91.4	148.1	772.90	0.953	95.3

#### Electrochemical impedance spectroscopy (EIS) measurements

**Figure 8:**
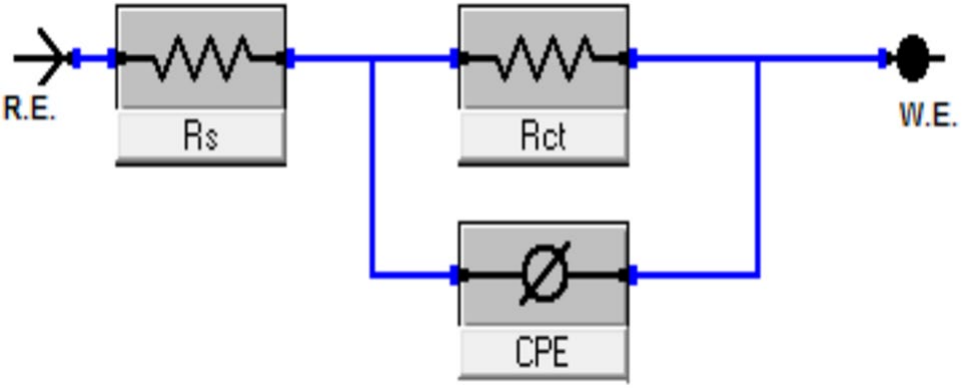
Electrical equivalent circuit model used to fit the impedance spectral data.

**Figure 9:**
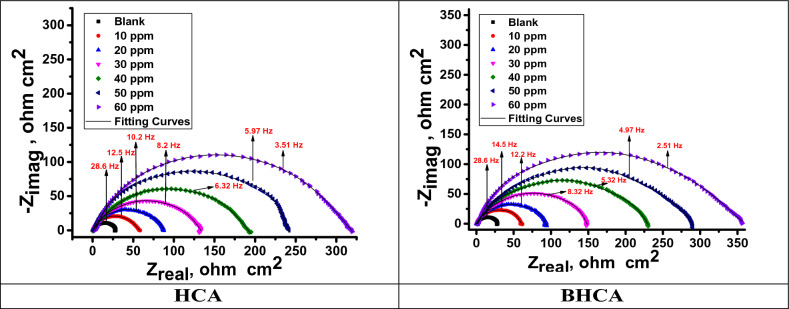
The Nyquist bends for dissolution of CS in 1 M HCl in the absence and with altered doses of HCA &BHCA at 298 K.

**Figure 10:**
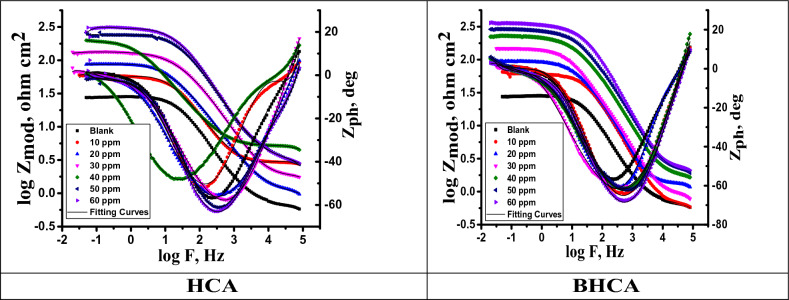
The Bode bends for corrosion of CS in 1 M HCl in the absence and attendance of altered doses of HCA & BHCA at 298 K.

Surface properties and mechanism of corrosion inhibition can be obtained and examined from EIS diagrams^31^. Figure 8 shows the electrical circuit model that corresponds to this system. It comprises of the charge transfer resistance (R_ct_) and the constant phase element (CPE), both of which are parallel combinations that are in series with the solution resistance (Rs). Altered electrochemical parameters are shown in Table 7. It may be inferred that the slower corrosion process is shown by the higher values of R_s_ in the HCl solution with HCA and BHCA compared to the blank^62^.To provide a more accurate match, CPE is reported rather than pure double layer capacitance because the double layer at the interface does not behave like an ideal capacitor. After that, the metal will begin to build a passive layer that tends to act as a shield for the link between Fe and the functional groups of the HCA and BHCA inhibitors that have been synthesized. The R_ct_ is bigger the denser the passive layer is compared to the value^63^. Figures 9 and 10 respectively; illustrate Nyquist and Bode graphs in the presence and absence of various concentrations of the derivatives HCA and BHCA inhibitors in 1 M HCl solution. The first incomplete circle shows how the addition of these chemicals caused a delay in the rate of charge transfer, while the other half circle shows how the heterogeneity of the surface is responsible for this^64^. Comparing the Bode charts (Fig. 10) to those of the free acid, their magnitude increased. This could be explained by the inhibitor molecules adhering to the surface^65^. The observation that the impact is concentration-dependent suggests that at greater inhibitor concentrations, the protective layer formed by the adsorbed additives will thicken and the inhibitory effect will be enhanced. By carefully reviewing Fig. 10, it was clear that the profile of all the plots into the hostile environment, both with and without inhibitors, did not actually alter the process of CS dissolution under study. As a result of the chloride ions being dissolved by BHCA and HCA, the diameter of the haves of the circles gradually raises with increased inhibitor dosages. An analogous circuit with charge transfer reaction is employed in Fig. 8 to assess the results from the EIS measurement. Data of the capacitance double layer (C_dl_) can be measured using the expression shown in Eq. (10)^66^ and the parameter CPE data Y_0_ and n:

10 $$C_{dl} = \, Y_{0} \left( {\omega_{max} } \right)^{n - 1}$$

“Where ω_max_ is the angular frequency at which the imaginary factor of the impedance arrives its maximum data, ω_max_ =2πf_max_, f_max_ is the maximum frequency,” n (n=α/(π/2, α is the phase angle)” is the deviance parameter of the CPE: − 1 ≤ n ≤ 1 and Y_o_ is magnitude of the CPE. The number n is a measure of the surface's roughness, and a rise in this number could indicate a reduction in the surface heterogeneity brought on by the adsorption of inhibitor molecules. Additionally, the Table shows that the n value varies directly with the inhibitor concentration; whereas Yo does the opposite. The dielectric constant is lowered as a result of the inhibitors adsorbed on the CS surface replacing some pre-adsorbed water molecules, which in turn lowers C_dl_^67^. The development of the inhibitor film may also contribute to the decline in C_dl_^68^. The chi-square **(χ**^**2**^**)** parameter, a measure of the model's goodness of fit, did not exceed 31x10^−3^, indicating a very high fit of the experimental impedance data to the electrical equivalent circuit. “The order of the prepared inhibitors based on IE% is BHCA>HCA”

**Table 7:** EIS data of CS in 1 M HCl in the absence and presence of altered doses of HCA and BHCA at 298 K.

Cpd	Conc. ppm	R_s_ Ω cm^2^	Y_o_ µΩ^−1^ s^*n*^ cm^−2^	n	C_dl_, μF cm^−2^	R_ct_ Ω cm^2^	θ	% IE	Goodness of fit (χ^2^)
Blank	–	1.514	578	0.986	549	46.20	–	–	19.78 × 10^−3^
HCA	10	2.184	390	0.990	376	81.70	0.435	43.5	14.39 × 10^−3^
	20	2.043	301	0.992	292	104.50	0.558	55.8	22.61 × 10^−3^
	30	2.815	214	0.993	208	134.20	0.656	65.6	31.64 × 10^−3^
	40	2.908	172	0.995	169	219.30	0.789	78.9	14.22 × 10^−3^
	50	2.914	129	0.995	127	348.10	0.867	86.7	26.25 × 10^−3^
	60	2.974	69	0.996	68	531.60	0.913	91.3	21.33 × 10^−3^
BHCA	10	1.345	382	0.984	361	87.60	0.473	47.3	21.16 × 10^−3^
	20	1.682	296	0.988	283	110.30	0.581	58.1	27.66 × 10^−3^
	30	1.807	209	0.990	201	142.70	0.676	67.6	25.78 × 10^−3^
	40	1.911	170	0.991	165	239.50	0.807	80.7	14.71 × 10^−3^
	50	1.937	122	0.994	119	361.40	0.872	87.2	25.47 × 10^−3^
	60	1.975	60	0.996	59	572.10	0.919	91.9	17.33 × 10^−3^

### Surface morphology

**Figure 11:**
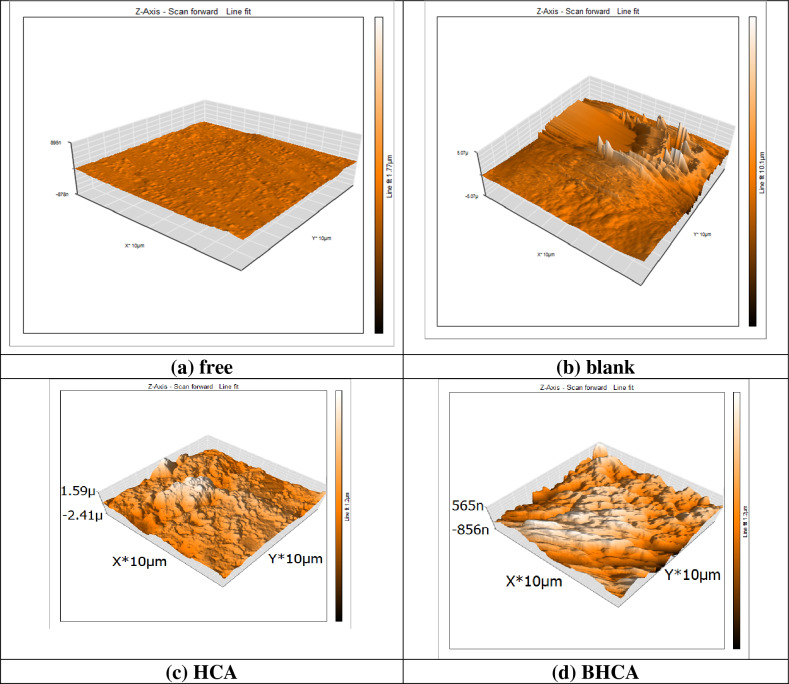
AFM spectra for (**a**) 3D image of free polished CS surface (**b**) 3D image of CS in 1 M HCl in absence of inhibitors (**c**) 3D image of CS in 1 M HCl in presence of 60 ppm of inhibitor HCA (**d**) 3D image of CS in 1 M HCl in presence of 60 ppm of inhibitor BHCA.

Three-dimensional topography measurements were mostly performed using an atomic force microscope. Figure 11a–d depicts the three-dimensional AFM images. A polished CS surface (roughness = 13.2 nm) is shown in Fig. 11a. A CS surface in 1 M HCl solution without inhibitor is shown in Fig. 11b (roughness = 568.3 nm). Figure 11c,d shows the CS surface with BHCA and HCA present, respectively. Figure 11c,d shows that the CS surface with HCA & BHCA inhibitors has significantly less damage than the CS surface dipped in 1 M HCl solution without inhibitor (Fig. 11b). It was calculated that the CS had an average roughness of 568.3 nm in 1 M HCl without inhibitor. The average roughness was reduced to 466.40 nm and 525.21 nm, respectively, in the presence of 60 ppm of BHCA and HCA, confirming the presence of inhibitors on the CS surface.

### Theoretical studies calculations

#### Quantum chemical calculations

**Figure 12:**
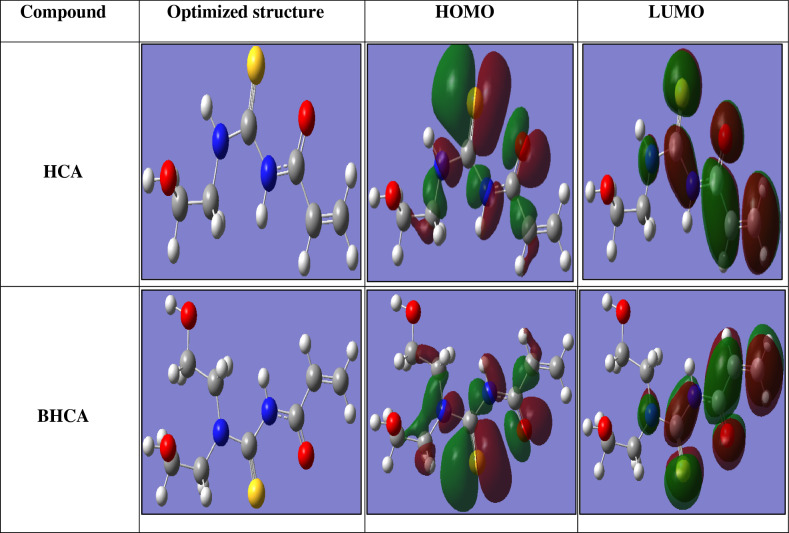
Molecular structure of the investigated composites, and its frontier molecular orbital density distribution (HOMO and LUMO) and the optimized molecular structure.

When value of the lowest unoccupied molecular orbital (E_LUMO_) is lower, “then the atom will have the higher tendency to accept electrons^47^. On the other hand, when the value of the highest occupied molecular orbital (E_HOMO_) of the inhibitor is higher, then it will be easier to offer electrons to the empty d-orbital of metal surface which central to the higher in its inhibition efficacy. “The calculated quantum chemical parameters derived from quantum calculations (E_HOMO_, E_LUMO_, μ, ΔE) of the prepared HCA and BHCA inhibitors shown in Table 8. The difference ΔE (Energy gap) = E_HOMO _− E_LUMO_ is the energy required to move an electron from HOMO to LUMO. Low ΔE facilitates adsorption of the molecule and thus will cause higher inhibition efficacy, as ΔE decreases, the reactivity of the molecule increases leading to increase the inhibition efficacy of the molecule^69^. Figure 12 shows molecular structure of investigated derivatives, and their frontier molecular orbital density distribution (HOMO and LUMO) while the values of quantum chemical parameters are shown in Table 8. The order of the prepared inhibitors based on IE% is BHCA > HCA.

**Table 8:** The quantum parameters for the investigated composite utilized (PM3).

Inhibitor	− E_HOMO,_ (eV)	− E_LUMO_, (eV)	Δ E, (eV)	Μ, (debye)
HCA	5.339	0.835	4.504	6.909
BHCA	5.044	0.739	4.305	7.256

#### Monte Carlo simulation studies

**Figure 13:**
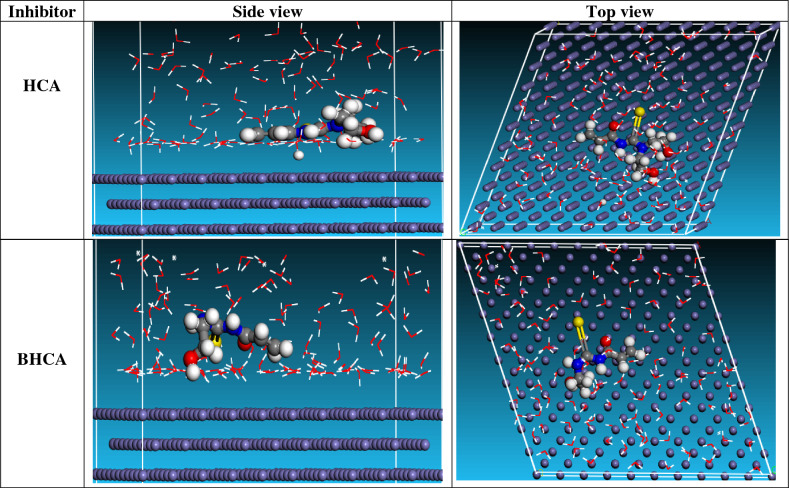
The inhibitors' equilibrium adsorption configurations on the surface of Fe (110): top and side views.

Low energy adsorption sites on both periodic and non-periodic substrates, as well as preferential adsorption of mixtures of adsorbate components, could represent the most stable adsorption sites on metal surfaces, and they could be defined using Monte Carlo simulations^70^. Different parameters derived from the Monte Carlo simulation shown in Table 9. Total energy of the substrate-adsorbate outline is contained in the parameters, measured in kJ mol^−1^. Adsorption energy is the result of adding stiff energy with deformation energy. The energy of the iron surface serving as the study's substrate is taken into account to be zero^71^. Additionally, the energy of adsorption in kJ mol^−1^ indicates the amount of energy released (or needed) when the relaxed adsorbate component is adsorbed on the substrate^72^. When the unrelaxed adsorbate components are adsorbed on the substrate, the energy of the stiff adsorption refers to the energy liberated (or needed), expressed in kJ mol^−1^. The deformation energy measures the energy released when the adsorbate constituents are relaxed on the substrate surface, expressed in kJ mol^−1^^73^. The energy of substrate-adsorbate configurations where one of the adsorbate components has been deleted is reported in Table 9 as (dE_ads_/dNi), which is expressed in kJ mol^−1^. HCA and BHCA inhibitors produced significant adsorption energy in negative values discovered throughout the modeling process, as indicated in Table 9. Great adsorption energy values indicate high inhibitory effectiveness. Equilibrium adsorption arrangements of the inhibitor compounds for HCA and BHCA in both top and side views are shown in Fig. 13 BHCA > HCA is the order of the produced inhibitors based on %IE.

**Table 9:** “Equilibrium adsorption configurations of the inhibitor molecules on the Fe (110) surface: side view and top view”.

Structures	Total energy	Adsorption energy	Rigid adsorption energy	Deformation energy	Compound dE_ads_/dNi	H_2_O dE_ads_/dNi
Fe (1 1 0)–HCA	3240.9	− 3143.3	− 3306.6	163.3	− 73.6	− 7.1
Fe (1 1 0)–BHCA	3248.6	− 3159.3	− 3321.2	161.8	− 150.1	− 7.3

### Corrosion inhibition mechanism

The main factor in the mechanism of the inhibition processes of the prepared HCA and BHCA corrosion inhibitors is the adsorption of the inhibitor molecules on the metal surface forming a protective layer preventing the metal suspension. The affinity of the inhibitor to be adsorbed on the metal surface is due to the presence of function groups capable to firmly anchor the inhibitor molecules on the metal surface (Supplementary information). The inhibition efficacy rises with increasing the inhibitor concentration which is attributed to the formation of a protective layer of inhibitor molecule at the metal/solution interface. The thickness of this layer increases by increasing the inhibitor concentration and thus increases its inhibition efficacy toward CS corrosion in 1 M HCl solution. As the temperature increases the inhibition efficacy decreases which indicate that the adsorption process is physisorption also it is revealed from electrochemical study using PDP measurements that the prepared inhibitors act as mixed type inhibitors that inhibit both anodic and cathodic reactions.

**Figure 14:**
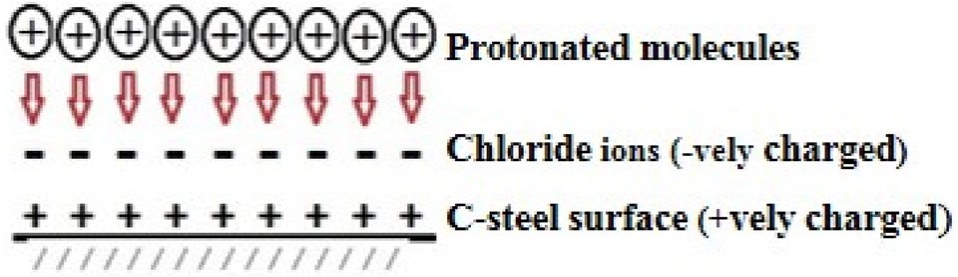
Schematic representation of inhibition mechanism.

Typically, two adsorption modalities could be taken into account. Through chemisorption, which involves displacing water molecules from the metal surface and sharing electrons between oxygen and iron, the neutral BHCA and HCA can be adsorbed on the metal surface. On the basis of donor–acceptor interactions between the p electrons of the heterocyclic and the unoccupied d-orbitals of iron, the BHCA and HCA molecules can also be adsorbed on the metal surface. The protonated BHCA or HCA in the acid medium may be adsorbed onto the metal surface (which bears −ve charge due to the adsorption of Cl^−^ ions on it) by electrostatic interactions between the positive molecules and already-adsorbed chloride ions, or due to the formation of metal complexes of Fe^2+^ and BHCA or HCA derivatives. These complexes might adhere to the surface of CS via the Vander Waals force to create a protective coating (Fig. 14).

## Conclusion

Two new inhibitors were synthesized, characterized, and evaluated as corrosion inhibitors for API 5L X52 CS in 1 M HCl in this study. To investigate the corrosion inhibition efficacy of the prepared HCA and BHCA corrosion inhibitors, ML, different electrochemical measurements, different theoretical studies, and the AFM technique to study surface morphology were used. The study revealed the following: The max inhibition efficacy of the prepared corrosion inhibitor was found to be 90.90 and 91.7% at 60 ppm for HCA and BHCA, respectively. Different electrochemical measurements especially PDP measurements showed that the prepared HCA and BHCA inhibitors act as mixed inhibitor. The adsorption of the inhibitors on metal surface obeyed Langmuir isotherm. The adsorption free energy of the inhibitors on the metal surface indicates the physisorption type of the inhibitor as the inhibition efficacy decreased with the increase of the temperature. Data obtained from different electrochemical and different theoretical studies are in good agreement with the values obtained from the ML measurements. The agreement between these independent techniques proves the validity of the results”. The order of the inhibitors based on the inhibition efficacy (%IE) is: BHCA > HCA.

## Supplementary Information

Supplementary Information.

## Data Availability

Authors can confirmed that all relevant data are included in the article.
