# Publisher Correction: Synthesis and characterization of novel acrylamide derivatives and their use as corrosion inhibitors for carbon steel in hydrochloric acid solution

**DOI:** 10.1038/s41598-023-39595-4

**Published:** 2023-08-01

**Authors:** A. S. Fouda, E. M. Khalil, G. A. EL-Mahdy, M. M. Shaban, A. S. Mohammed, N. A. Abdelsatar

**Affiliations:** 1 Deparment of Chemistry, Faculty of Science, Mansoura University, Mansoura, 35516 Egypt; 2 Department of Chemistry, Faculty of Science, Helwan University, Cairo, Egypt; 3 Applied Surfactant Lab, Egyptian Petroleum Research Institute, Nasr City, Cairo Egypt; 4 Refining and Processing Deputy, Egyptian General Petroleum Corporation, Cairo, Egypt; 5 Department of Chemistry, Faculty of Science, Ain Shams University, Cairo, Egypt

Correction to: *Scientific Reports* https://doi.org/10.1038/s41598-023-30574-3, published online 02 March 2023

The original version of this Article contained errors in Equations 7 and 8.

In the original version of this article, the value “55.5” was incorrectly placed as a denominator, where

$${K}_{ads}=1/55.5exp \;[-\Delta {G}_{ads}^{o}/RT]$$

now reads:

$$55.5 {K}_{ads}=exp \;[-\Delta {G}_{ads}^{o}/RT]$$

The original Article has been corrected.

Additionally, the original PDF version of this Article contained errors in Equation 8, where the minus sign is missing:

$${k}_{corr}=A \;exp \;({E}_{a}^{*}/RT)$$

now reads:

$${k}_{corr}=A\; exp \;(-{E}_{a}^{*}/RT)$$

This error has now been corrected in the PDF version of the Article; the HTML version was correct from the time of publication.

