# Supplementary material for: Synthesis and characterization of novel acrylamide derivatives and their use as corrosion inhibitors for carbon steel in hydrochloric acid solution
Source: Sci Rep. 2023 Mar 2;13:3519. doi: 10.1038/s41598-023-30574-3 (PMC9981741; doi:10.1038/s41598-023-30574-3)
Supplement: Supplementary file 1 — Supplementary Information. [file 41598_2023_30574_MOESM1_ESM.docx]

**General method for preparation of inhibitors:**

1. Reaction of acryloyl chloride (0.9 g., 100 mmol) with ammonium thiocyanate (0.76 g., 100 mmol)in dry acetone with stirring for 10 minutes, furthermore, the ammonium chloride was filtered of and the reaction mixture stirred with different amines namely Diethanol amine and ethanol amine (100 mmol.) respectively for 30 minutes, then acetone was evaporated and wash the product with petroleum ether to remove the unreacted compounds the remaining oil is the product (**BHCA**) and (**HCA**) respectively
2. **Preparation of Inhibitor (BHCA)**

**N-(bis(2-hydroxyethyl)carbamothioyl)acrylamide**

Dark brown liquid, 82% yield, IR (KBr, ν /cm^-1^): broad band located at 3336.9 cm^-1^ (*ν*_OH),_ 3336.9 cm^-1^  (*ν*_NH_); 1704.6 cm^-1^ (*ν*_C=O_) **( SF1).** ^1^H-NMR (300 MHz, DMSO-d6) figure (4.2): δ 2.0 (s, 1H, NH, D2O exchangeable), 2.4 (t, 4H, H next to OH), 2.5 (dd, 1H, CH(b)), 2.9 (t, 4H, H next to N), 3.1 (q, 1H, CH(a)), 3.6 (dd, 1H, CH(c)), 6.2 (s, 2H, 2OH, D2O) exchangeable **(SF2).**

**SF1: I.R spectra for compund (BHCA)**


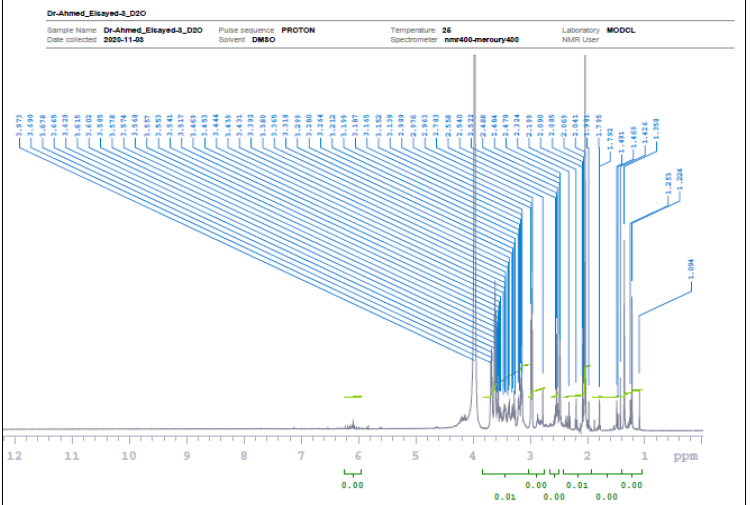


**SF2: NMR spectra for compund (BHCA)**

1. **Preparation of Inhibitor (HCA)**

**N-((2-hydroxyethyl) carbamothioyl) acrylamide**

Light brown liquid, 89% yield, IR (KBr, ν /cm-1) figure (4.3): broad band located at 3281..8 cm^-1^ (νOH), 3281.8, 3120.5 cm^-1^ (νNH); 1690.2 cm^-1^ (νC=O) **(SF3).** 1H-NMR (300 MHz, DMSO-d6) figure (4.4): δ 2.2 (t, 2H, H next to OH), 2.5 (t, 2H, H next to N), 3.5 (dd, 1H, CH(b)), 4.2 (q, 1H, CH(a)), 6.2 (dd, 1H, CH(c)), 7.8 (s, 1H, NH, D2O exchangeable), 8.0 (s, 1H, NH next to CO, D2O exchangeable), 8.1 (s, 1H, 1OH, D2O exchangeable) **(SF4).**

**SF3: I.R spectra for compund (HCA).**


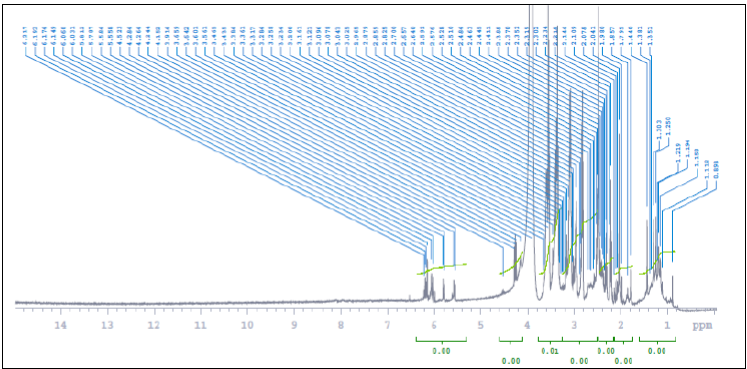


**SF4: NMR spectra for compund (HCA) Discussion:**

Our target is how to prepare a green corrosion inhibitor which can be prepared in industrial scale, this target was obtanid from reaction of [acryloyl chloride](https://www.google.com/search?sxsrf=AJOqlzXmfs4CDp4cSKOqdhSbEMeAXUFMbQ:1675361297206&q=acryloyl+chloride&spell=1&sa=X&ved=2ahUKEwiQ4_WXt_f8AhXBV6QEHblKDZQQkeECKAB6BAgJEAE) with ammonium thiocyanate in dry acetone with stirring add different amines namely diethanol amine and ethanol amine to afford (BHCA), (HCA) respectively. The structure was proved by finding *ν*_OH at_ 3336.9 cm^-1 ,^ *ν*_NH_ 3336.9 cm^-1^  and *ν*_C=O_ 1704.6 cm^-1^ for the compound (BHCA) and presence of two OH and NH in NMR at δ 6.2 and 2.0 respectively , while we found two *ν*_NH_ at 3281.8, 3120.5 cm^-1^ and ν_OH_ at 3281..8 cm^-1^ , furthermore, in NMR we find one OH only at δ 8.1 while we have two NH at δ 7.8 and 8.0 this improve the structure of the compound **(HCA).**
